# Synthesis, Characterization and Biological Evaluation of Transition Metal Complexes Derived from N, S Bidentate Ligands

**DOI:** 10.3390/ijms160511034

**Published:** 2015-05-15

**Authors:** Enis Nadia Md Yusof, Thahira Begum S. A. Ravoof, Edward R. T. Tiekink, Abhimanyu Veerakumarasivam, Karen Anne Crouse, Mohamed Ibrahim Mohamed Tahir, Haslina Ahmad

**Affiliations:** 1 Department of Chemistry, Faculty of Science, Universiti Putra Malaysia, UPM Serdang 43400, Malaysia; E-Mails: enisnadia89@gmail.com (E.N.M.Y.); abhi.veerakumarasivam@gmail.com (A.V.); kacrouse@gmail.com (K.A.C.); ibra@upm.edu.my (M.I.M.T.); haslina_ahmad@upm.edu.my (H.A.); 2 Department of Chemistry, University of Malaya, Kuala Lumpur 50603, Malaysia; E-Mail: edward.tiekink@gmail.com; 3 Medical Genetics Laboratory, Faculty of Medicine and Health Sciences, Universiti Putra Malaysia, UPM Serdang 43400, Malaysia; 4 Department of Chemistry, Cape Breton University, Sydney, NS B1P 6L2, Canada

**Keywords:** bidentate NS ligands, crystal structure analysis, hydrogen bonding, cytotoxic activity, DNA binding

## Abstract

Two bidentate NS ligands were synthesized by the condensation reaction of S-2-methylbenzyldithiocarbazate (S2MBDTC) with 2-methoxybenzaldehyde (2MB) and 3-methoxybenzaldehyde (3MB). The ligands were reacted separately with acetates of Cu(II), Ni(II) and Zn(II) yielding 1:2 (metal:ligand) complexes. The metal complexes formed were expected to have a general formula of [M(NS)_2_] where M = Cu^2+^, Ni^2+^, and Zn^2+^. These compounds were characterized by elemental analysis, molar conductivity, magnetic susceptibility and various spectroscopic techniques. The magnetic susceptibility measurements and spectral results supported the predicted coordination geometry in which the Schiff bases behaved as bidentate NS donor ligands coordinating via the azomethine nitrogen and thiolate sulfur. The molecular structures of the isomeric S2M2MBH (**1**) and S2M3MBH (**2**) were established by X-ray crystallography to have very similar l-shaped structures. The Schiff bases and their metal complexes were evaluated for their biological activities against estrogen receptor-positive (MCF-7) and estrogen receptor-negative (MDA-MB-231) breast cancer cell lines. Only the Cu(II) complexes showed marked cytotoxicity against the cancer cell lines. Both Schiff bases and other metal complexes were found to be inactive. In concordance with the cytotoxicity studies, the DNA binding studies indicated that Cu(II) complexes have a strong DNA binding affinity.

## 1. Introduction

Despite only a small difference in the backbone of their structure, Schiff bases having N and S donor atoms have been shown to possess a wide spectrum of biological activities and physicochemical properties [1,2], such as metal complexation, electrochemical, adsorptive and crystallographic properties [3,4]. Schiff bases that contain an imino group (–RC=N–) are formed by the condensation of a primary amine with an active carbonyl group. A number of Schiff bases containing the imino functionality have been shown to have a wide range of biological activities, including antibacterial, antifungal, antidiabetic, antitumor, antiproliferative, anticancer, anticorrosive and anti-inflammatory activities [5,6,7,8]. It is believed that the biological activity is related to the hydrogen bonding through the imino group of Schiff bases with the active centers of the cell constituents [9]. Ejiah *et al.*, (2013) reported that the anti-bacterial activity was dependent on the position of substituents in the benzene ring, type of bacteria (whether Gram-positive or Gram-negative) and also the solvent used in the experiment. Gram-positive bacteria had higher MIC values and hence, were less active when compared to Gram-negative bacteria. This could be due to thick peptidoglycan cell wall of Gram-positive bacteria that restricts penetration or diffusion of the compound [10].

Schiff bases and their metal complexes have been shown to be promising leads for both synthetic and structural research due to their relatively simple synthesis and structural diversity [11]. It has also been reported that metal complexes were more biologically active as compared to noncoordinated Schiff bases [12,13,14]. Chelation to the metal ion can be useful in in developing cytotoxic drugs, radioactive agents in imaging studies, and radio immunotherapy (RIT) [15]. Marzano *et al.*, (2006) reported that tris-(hydroxymethyl)phosphine copper(I) complexes containing the new bis(1,2,4-triazol-1-yl)acetate ligand showed greater *in vitro* antitumor activity as compared to the Schiff bases alone. These complexes were able to overcome cisplatin resistance by triggering paraptosis, a non-apoptotic mechanism of cell death in the resistant cells [16].

Many chemotherapeutic drugs have been designed based on the ability of these synthetic drugs to target the DNA molecule. A new bidentate Schiff base derived from 2,4-dihydroxybenzophenone and aniline and its metal complexes were synthesized and evaluated for their DNA binding ability and the *in vitro* biological activity of these complexes were markedly better than that of the Schiff base. The results indicated that Cu(II) complexes bound to the DNA through noncovalent interactions [17]. Sunscreen creams are commonly produced from 2-hydroxy-4-methoxybenzophenone and 2-hydroxy-4-methoxy-4-methoxybenzophenone. These creams help to avoid photosensitization, phototoxicity or allergic reactions from various treatments [18]. Based on the DNA binding studies involving copper and nickel complexes derived from this Schiff base, it was found that the complexes showed moderate DNA binding and oxidative cleavage activities. The complexes interacted with DNA through the stacking interactions between the aromatic chromophore and the base pairs of DNA. It was concluded that the Schiff base and in particular, their metal complexes have efficient bio-efficacy, DNA binding and cleavage ability [17].

Although cisplatin is widely-used in cancer therapy, it is associated with a spectrum of side effects, such as anemia, diarrhea, alopecia, petechia, fatigue, nephrotoxicity, emetogenesis, ototoxicity, and neurotoxicity [17]. These side-effects limit the full efficacy of this potent drug. Thus, research involving the development of new metal-based anticancer drugs with minimal side effects and maximal curative potential has intensified over the years. In this study we synthesized and characterized dithiocarbazate Schiff bases and their metal complexes. We also evaluated the cytotoxicity and binding activity of these complexes for their potential as anti-cancer compounds.

## 2. Results and Discussion

### 2.1. Synthesis

**Scheme 1:**
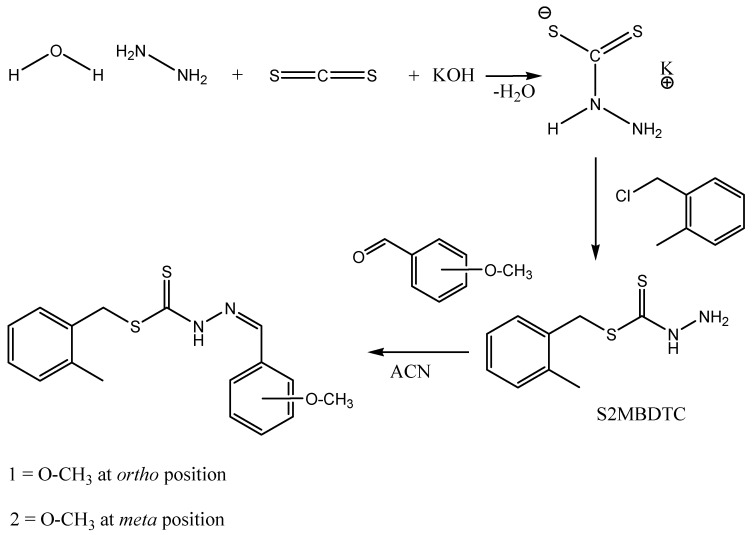
Synthetic pathway of **1** and **2**.

The Schiff bases (**1**) and (**2**) were prepared by the condensation reaction of S2MBDTC and 2-methoxybenzaldehyde (**1**) or 3-methoxybenzaldehyde (**2**) in acetonitrile with 72%–75% yields (Scheme 1). Two moles of Schiff bases were then reacted with 1 mol of various metal salts in an ethanol-acetonitrile solution in the ratio of 2:1 to produce the metal complexes (**3**–**8**). Compounds **1**–**8** are non-hygroscopic, stable at room temperature, and soluble in common organic solvents, especially dimethyl sulfoxide (DMSO) and dimethyl furan (DMF). The melting points were sharp (over 1 or 2 °C) indicating that the complexes were free of impurities. The chemical properties of both Schiff bases (**1**) and (**2**) and their metal complexes (**3**–**8**) were characterized by elemental analysis, IR, UV-Vis, ^1^H-NMR, ^13^C-NMR, mass spectroscopy and Single X-ray diffraction analysis (only for compounds **1** and **2**). Table 1 shows that the percentage of carbon, hydrogen, nitrogen, and metal were in good agreement with the proposed formulae. The data achieved were in agreement with the proposed structures. The molar conductivity data for the metal complexes **3**–**8** showed that the complexes are non-electrolytes in dimethyl sulfoxide (DMSO) where the molar conductivity values fall in the range of 0.28–2.44 Ω^−1^·cm^2^·mol^−1^, which is well below 25 Ω^−1^·cm^2^·mol^−1^, the amount beyond which the complexes are considered 1:1 electrolytes [19].

**Table 1:** Analytical data of the Schiff bases and their metal complexes

Serial Number	Compound	% Found (Calculated)			
		C	H	N	M
**1**	S2M2MBH	62.51 (61.79)	5.44 (5.49)	8.00 (8.48)	-
**2**	S2M3MBH	64.06 (61.79)	5.50 (5.49)	9.08 (8.48)	-
**3**	[Cu(S2M2MB)_2_]	57.18 (56.52)	4.80 (4.74)	8.20 (7.75)	8.40 (8.8)
**4**	[Ni(S2M2MB)_2_]	56.55 (56.91)	4.82 (4.78)	8.21 (7.81)	8.54 (8.18)
**5**	[Zn(S2M2MB)_2_]	57.34 (56.38)	4.80 (4.73)	8.24 (7.74)	9.02 (9.03)
**6**	[Cu(S2M3MB)_2_]	56.32 (56.52)	4.58 (4.74)	7.94 (7.75)	8.30 (8.8)
**7**	[Ni(S2M3MB)_2_]	56.96 (56.91)	4.76 (4.78)	8.21 (7.81)	8.51 (8.18)
**8**	[Zn(S2M3MB)_2_]	55.46 (56.38)	4.65 (4.73)	7.88 (7.74)	8.79 (9.03)

### 2.2. ^1^H- and ^13^C-NMR Spectra

Table 2 shows the ^13^C-NMR and ^1^H-NMR spectral data for compounds **1** and **2**. The ^13^C-NMR spectra of compounds **1** and **2** showed the presence of deshielded –HN–C(=S)S at 196.62 and 196.08 ppm. The positions of these chemical shifts proved that **1** and **2** predominates as the thione tautomer in DMSO-*d*_6_ solution. The –CH_2_, which is located in between the CSS group and benzene ring, was assigned at 36.84 and 38.04 ppm, was shielded due to the proximity of electronegative residues close to the CH_2_ group. The free CH_3_ attached to the benzene ring for **1** and **2** was shielded by the aromatic group and the signal was found ranging between 19.40–19.41 ppm. Resonances attributed to the carbon nuclei in the aromatic rings of the Schiff bases were observed in the region of 112.53–147.13 ppm, comparable to that previously reported by Dilović *et al.* [20]. The assignment for resonance peaks of carbon atoms in the aromatic rings were confirmed by DEPT NMR and all the signals matched with the proposed structure. The signal that appeared closest to the septet splitting of the solvent indicated the presence of the methoxy carbon atom, which fell between 55.74 and 55.88 ppm. The methylene carbon is less shielded as it is closely bonded to the electronegative oxygen atom. Apart from that, the Schiff bases exhibited a –CH resonance near the CSS group at 158.85 and 161.98 ppm, which was deshielded due to the electronegative effect experienced by the carbon atom.

The ^1^H-NMR spectra of compounds **1** and **2** displayed chemical shifts at 13.29 and 13.22 ppm indicating the presence of a proton that was directly attached to the N atom adjacent to the –C=S and –N=C corresponding to an *sp*-type proton appearing as a singlet. This proton signal appeared deshielded due to the close proximity to the electronegative nitrogen atom. The presence of the methylene (–CH_2_) proton in between the sulfur atom and the benzene ring was clearly observed at 4.38 ppm. The singlet due to a deshielded –CH proton was observed at 7.70 and 8.30 ppm. The –CH proton occurred at the deshielded area due to the electron repulsion from the neighboring electronegative sulfur atom, which was located near to the proton. There was one singlet appearing at 2.29 and 2.30 ppm corresponding to an sp^3^-type of proton (–CH_3_), which is located at the *ortho* position attached to the benzene ring of the dithiocarbazate backbone, which was shielded by the benzene ring. The methyl proton of the methoxy group (O–CH_3_) was also clearly present at 3.80 ppm (**1**) and 3.74 ppm (**2**). The resonances corresponding to the aromatic protons were observed at chemical shifts 6.92–7.70 ppm. The integration values from the ^1^H-NMR spectra for **1** and **2** matched exactly with the number of hydrogens proposed for the respective structures.

**Table 2:** ^13^C-NMR and ^1^H-NMR spectral data for compounds **1** and **2**.

Compound	^13^C-NMR Assignment, δ (ppm)						^1^H-NMR Assignment, δ (ppm)				
	–C=S	–CH_3_	–CH_2_	–CH	O–CH_3_	Aromatic Carbons	NH	CH	CH_2_	CH_3_	Aromatic Protons
**1**	196.6	19.4	38.0	158.9	55.7	112.5–142.8	s, 1H (13.3)	s, 1H (7.7)	s, 2H (4.4)	s, 3H (3.8 & 2.3)	d, 1H (7.2, 7.3, 7.7, 7.1) t, 1H (7.1, 7.1, 7.4, 7.0)
**2**	196.1	19.4	36.8	162.0	55.9	115.1–147.1	s, 1H (13.2)	s, 1H (8.3)	s, 2H (4.4)	s, 3H (3.7 & 2.3)	s, 1H (8.1) d, 1H (7.0, 7.2, 7.3, 7.6) t, 1H (7.1, 7.1, 7.1)

### 2.3. IR Spectra

The IR spectra provide information about any functional groups present in the molecules that support the successful formation of Schiff bases and metal complexes. Table 3 shows the comparative analysis of IR spectra of the Schiff bases (**1** and **2**) and metal complexes (**3**–**8**). Strong bands corresponding to aldehydic *v*(C=O) disappeared in the infrared spectra of compounds **1** and **2** indicating that **1** and **2** were successfully formed through condensation. The stretching vibrations of *v*(N–H), at 3085 and 3099 cm^−1^ were not present in the metal complexes (**3**–**8**). This is due to the deprotonation of the nitrogen atom upon complexation. Compounds **1** and **2** displayed sharp peaks at 1598 and 1609 cm^−1^, which corresponded to *v*(C=N), attributable to the azomethine group. This stretching band shifted to lower wavenumbers upon complexation to the metal ions [21]. In this work, the *v*(C=N) decreased upon complexation, indicating the coordination of compounds **1** and **2** to the metal ions via the azomethine nitrogen [22]. The shifting of hydrazinic *v*(N–N) band to both higher and lower wavenumbers in the infrared spectra of complexes **3**–**6** showed evidence of metal coordination through the azomethine nitrogen atom, as this was related to the reduction in the repulsion between lone pairs of electrons on the nitrogen atoms upon complexation [23,24,25]. Compounds **1** and **2** exist only in the thione tautomeric form due to the presence of strong *v*(C=S) bands at 736 and 775 cm^−1^ which did not appear in the spectra **3**–**8**, an observation that was in agreement with the NMR data. The splitting of *v*(CSS) of compounds **1** and **2** were found in the range of 729–770 cm^−1^. This was strong evidence to support that the coordination of the compounds **1** and **2** to the metal ions was via the thiolate sulfur, with the loss of a proton.

**Table 3:** Infrared, magnetic susceptibility and electronic spectral data of compounds 1–8.

Compound	IR Bands (cm^−1^)				µ_eff_ (BM)	Electronic Spectra (in DMSO) λ_max_(log ε_max_)
	*v*(NH)	*v*(C=N)	*v*(N–N)	*v*(C=S)/*v*(C–S)		
**1**	3085 (w)	1598 (m)	1035 (s)	736 (s)	-	352(4.86)
**2**	3099 (w)	1606 (m)	1043 (s)	775 (s)	-	341(4.63)
**3**	-	1584 (m)	1014 (s)	750 (s)	1.60	352(4.93); 601(2.57)
**4**	-	1579 (m)	1011 (s)	732 (s)	Diamagnetic	354(4.90); 609(2.08)
**5**	-	1593 (m)	947 (s)	729 (s)	Diamagnetic	354(5.21)
**6**	-	1573 (m)	964 (s)	732 (s)	1.67	624(2.23); 422(3.92); 324(4.61)
**7**	-	1573 (m)	969 (s)	770 (s)	Diamagnetic	342(4.88); 444(3.55); 609(2.07)
**8**	-	1599 (m)	957 (s)	731 (s)	Diamagnetic	341(5.08)

s = strong; m = medium; w = weak.

### 2.4. Magnetic Susceptibility and Electronic Spectral Studies

In order to obtain further structural information on the metal complexes, the magnetic moments and electronic spectra were measured and the results tabulated in Table 3. At room temperature, complexes **3** and **6** showed magnetic moments of 1.60 and 1.67 Bohr magneton (B.M), respectively, indicating one unpaired electron with the electronic configuration of d^9^. Complexes **3** and **6** are hence expected to have a square planar geometry, which is the predicted geometries of a metal ion with an unpaired electron at d_x_^2^_-y_^2^ orbital with magnetic moments of around 1.73 B.M [26]. Compound **4**, **5**, **7**, and **8** showed diamagnetic properties, again indicative of a four-coordinate geometry. The Ni(II) complexes, **4** and **7** have a d^8^ electronic configuration with a low spin state which commonly adopts a four-coordinated environment around the metal ion [27]. The diamagnetic Zn(II) complexes are expected to be four-coordinated and have a distorted tetrahedral structure. However, to date, we were unable to obtain suitable crystals of the metal complexes for single X-ray diffraction analysis.

The electronic spectral data of all the compounds is displayed in Table 3. In common transition metal complexes, the color consequences of light absorption is due to the transfer of electrons from an orbital primarily on the ligand to one primarily located on the metal ions, such as ligand-to-metal charge transfer (LMCT) or *vice versa*, metal-to-ligand charge transfer (MLCT). The yellow compounds **1** and **2** show intense intraligand absorptions in the range of 352 and 341 nm due to the π → π* transition, corresponding to the nonbonding electron pairs of the azomethine bond. Bands at 324–352 nm corresponded to a intraligand π → π* transition, whereas bands at 422 nm corresponded to an LMCT transition and bands at 601–624 nm corresponded to d-d band transitions which is a ^2^E_g_ → ^4^A_2g_. The high intensity π → π* transition overlapped with the LMCT transitions, thus the LMCT bands did not appear in some cases. The occurrence of a S→Cu LMCT band around 422 nm is common in square planar dithiocarbazate Cu(II) complexes [28,29]. All the Cu(II) complexes displayed a weak d-d transition, which is a spin-forbidden transition hence, the relative weakness of d-d band assigned as tetrahedral and square planar complexes. The Ni(II) complexes displayed absorption bands in the range of 342–354, 444 and 609 nm, due to the π → π* transitions, LMCT transitions, and d-d transitions, respectively. The three d-d bands corresponding to ^1^A_1g_ → ^1^A_2g_, ^1^A_1g_ → ^1^B_1g_, and ^1^A_1g_ → ^1^E_1g_ transitions are indicative of square planar Ni(II) complexes [18,27]. The magnetic moments for all the Ni(II) complexes further supported the proposed square planar configuration.

The electronic spectra of the yellow Zn(II) complexes only showed a band in the region 341–354 nm, due to the presence of LMCT transition. An electron migrates between an orbital that is predominantly ligand in character to an orbital that is predominantly zinc in character The LMCT transition of Zn(II) complexes were identified by their high intensity and the sensitivity of their energies to the solvent polarity.

### 2.5. Crystal Data and Molecular Structures

#### 2.5.1. Molecular Structures

**Figure 1:**
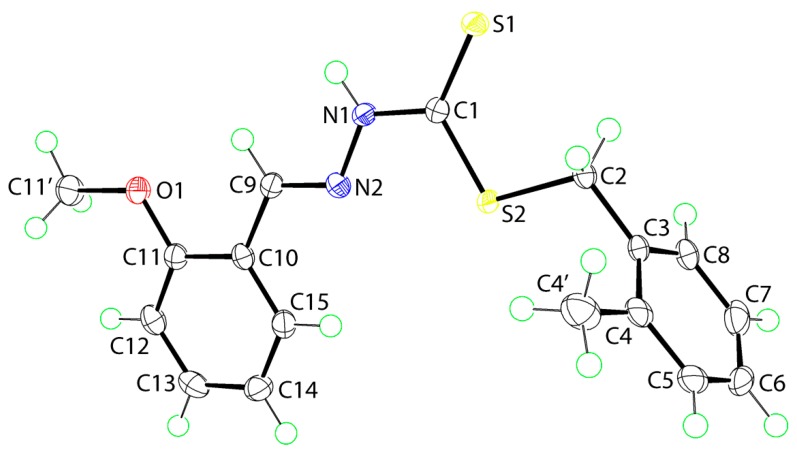
The molecular structure of **1** showing the atom-labeling scheme and 70% displacement ellipsoids.

Crystal structures were obtained for isomeric **1** and **2**. The molecular structure of **1** is shown in Figure 1 and selected geometric parameters are collected in Table 4. The central CN_2_S_2_ atoms are co-planar with a root mean square (r.m.s) = 0.014 Å. The adjacent tolyl ring is twisted out of this plane as seen in the value of the dihedral angle of 82.70(4)° but the methoxy-substituted ring is almost co-planar with the central residue forming a dihedral angle of 6.14(5)°. This implies the terminal rings are almost orthogonal. Indeed the dihedral angle between the rings is 87.05(5)° so that overall the molecule adopts the shape of the letter L. The methoxy group is co-planar with the ring to which it is attached as seen in the value of the C11'‒O1‒C11‒C12 torsion angle of 4.4(2)°. Thus, with the exception of the tolyl-methyl group, to a first approximation the molecule has mirror symmetry with the *ipso*- and *para*-carbon atoms of the tolyl group lying on the putative plane. The thione-S1 and amino-H atoms are *syn*, an important consideration for the crystal packing as discussed in *2.5.2*. The C1‒S1 bond distance is significantly shorter than the C1‒S2 bond, which, in turn is shorter than the C2‒S2 bond (Table 1), indicating significant double bond character in the thione C1‒S1 bond. The bond lengths of N1‒N2 and C9‒N2 are consistent with single and double bonds, respectively, so that despite the planarity in the central residue, there is little evidence for extensive delocalization of π-electron density over these atoms; the conformation about the C9‒N2 double bond is *E*. The angles subtended at the C1 atom by the thione-S1 atom are systematically wider than the S2‒C1‒N1 bond, again consistent with the double character of the thione C1‒S1 bond.

**Table 4:** Selected geometric parameters (Å, °) for **1** and **2**.

Parameter	1	2
C1‒S1	1.6613(14)	1.6724(14)
C1‒S2	1.7587(14)	1.7537(14)
C2‒S2	1.8245(14)	1.8241(15)
C1‒N1	1.3470(17)	1.3373(19)
N1‒N2	1.3694(16)	1.3765(17)
C9‒N2	1.2821(19)	1.2797(19)
S1‒C1‒S2	124.77(8)	124.68(9)
S1‒C1‒N1	122.02(10)	121.68(11)
S2‒C1‒N1	113.21(10)	113.64(11)
C1‒N1‒N2	118.76(11)	119.64(12)
N1‒N2‒C9	116.56(12)	115.74(12)

**Figure 2:**
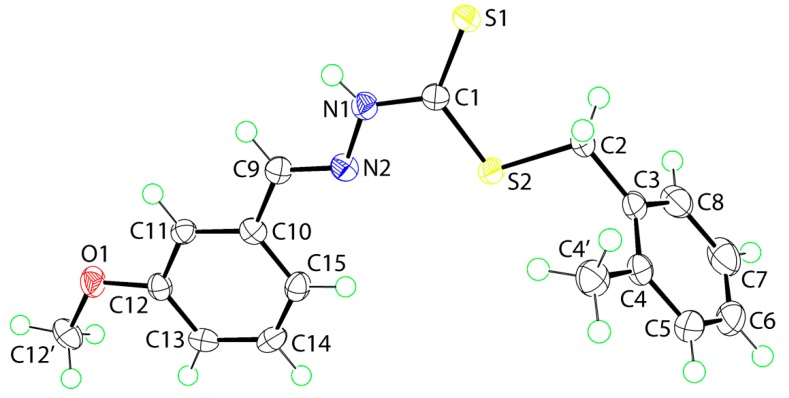
The molecular structure of **2** showing the atom-labeling scheme and 70% displacement ellipsoids.

The molecular structure of **2** is shown in Figure 2. To a first approximation, the structure of the 3-methoxy isomer is the same as the 2-methoxy isomer in **1**. The r.m.s. deviation for the central CN_2_S_2_ atoms is 0.010 Å, and this forms dihedral angles of 80.01(3) and 1.58(7)° with the tolyl and methoxyphenyl rings, respectively. The dihedral angle between the rings is 81.19(4)° consistent with an L-shape. The methoxy group is co-planar with the ring to which it is attached (C12'‒O1‒C12‒C11 = 179.28(13)°) and again, with the exception of the tolyl-methyl group, the entire molecule approximates mirror symmetry as for **1**. The anti-disposition of the thione-S1 and amine-H atoms, the *E* configuration about the C9‒N2 bond and the key geometric parameters conform to that discussed for **1**.

**Figure 3:**
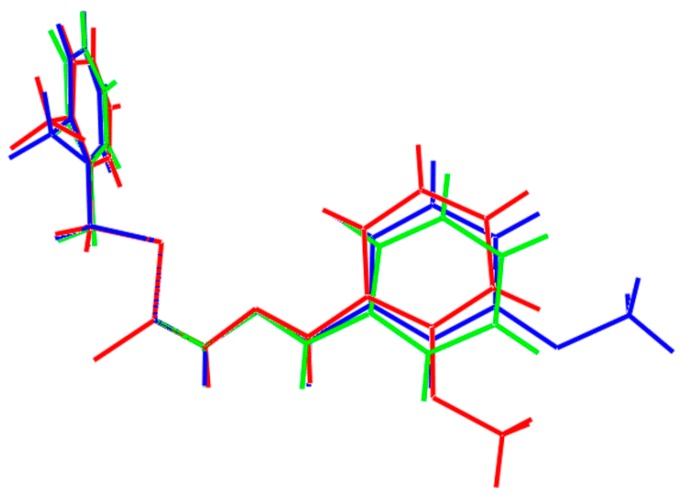
Overlay diagram of the molecular structures of **1** (red image), **2** (blue) and the unsubstituted parent compound (green) with the CS_2_ residues coincident.

The similarity between the molecules is highlighted in the overlay diagram shown in Figure 3, where the CS_2_ residues are coincident. Also included is the structure of the unsubstituted parent compound, *i.e.*, PhC(H)=NNC(=S)SCH_2_Ph [30]. This, too, has the same features as **1** and **2** indicating that substitution in the rings has not resulted in a significant difference in molecular conformation.

#### 2.5.2. Supramolecular Architectures

**Figure 4:**
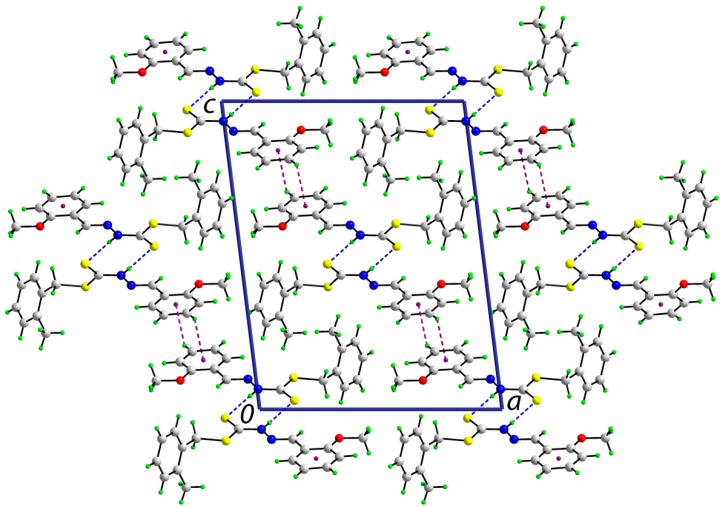
A view in projection down the *b*-axis of the unit cell contents of **1**. The N‒H···S and C‒H···π interactions are indicated by blue and purple dashed lines, respectively.

The *syn* disposition of the thione-S1 and amino-H atoms enables the formation of the cyclic eight-membered {···HNCS}_2_ thioamide synthon in the crystal structures of each of **1** and **2**; geometric parameters characterizing the intermolecular interactions are listed in Table 5. In **1**, the dimeric aggregates stack in columns along the *b*-axis. The only other intermolecular interactions within the standard criteria [31] are of the type C‒H···π, where the donor and acceptors are derived from the methoxyphenyl ring, and these serve to link the dimers into supramolecular layers parallel to [30]. A view of the unit cell contents is shown in Figure 4.

**Table 5:** Geometric parameters characterizing the key intermolecular contacts in **1** and **2**.

A	H	B	H···B (Å)	A···B(Å)	A-H-B(°)	Symmetry Operation
**1**						
N1	H1n	S1	2.503(14)	3.3665(12)	172.6(13)	1-*x*, 3-*y*, 1-*z*
C13	H13	Cg(C10–C15)	2.77	3.5374(15)	139	½- *x*, -½+*y*, 1½-*z*
**2**						
N1	H1n	S1	2.53(2)	3.3688(15)	168.5(19)	-*x*, -*y*, -*z*
C11	H11	O1	2.44	3.351(2)	160	-*x*, -½+*y*, ½-*z*
C12'	H12b	S1	2.84	3.5574(17)	130	*x*, 1½-*y*, ½+*z*
C12'	H12c	Cg(C10–C15)	2.80	3.6949(19)	152	*x*, 1½-*y*, ½+*z*

**Figure 5:**
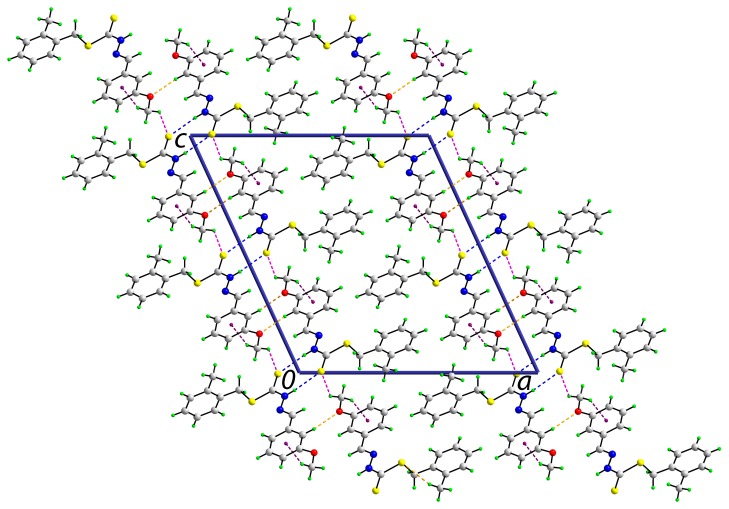
A view in projection down the *b*-axis of the unit cell contents of **2**. The N‒H···S and C‒H···π interactions are indicated by blue and purple dashed lines, respectively.

The centrosymmetric dimeric aggregates formed via {···HNCS}_2_ synthons also stack in columns parallel to the *b*-axis in the crystal structure of **2**. They are also assembled into layers that stack along the *a*-axis (Figure 5). The interactions between the dimers are of the type C‒H···O, C‒H···S and C‒H···π, with all donors and acceptors (except the thione-S1 atom) derived from the methoxyphenyl ring (Table 5).

### 2.6. Cytotoxic Activity

Compound **1**–**8**, standard drug (Tamoxifen), and dimethyl sulfoxide (DMSO) solvent were evaluated separately for their cytotoxic activity against MDA-MB-231 and MCF-7 cell lines using the MTT Assay method. Except for the Cu(II) complexes, the other test compounds did not show any activity against both cell lines. Complexation with Cu(II) ions also significantly reduced the polarity of the metal ions and enhanced the lipophilic character of central the Cu(II) atom. The complexation may facilitate faster diffusion through biological membranes [32] and also disturb the respiration process of the cells and thus restrict further growth of the organism [33,34]. These factors enhance the cytotoxic properties of Cu(II) complexes presumably by blocking the enzymatic activity of the cell or catalyzing cytotoxic reactions [35]. Hence, upon complexation, the cytotoxic activity of the Cu(II) complexes was drastically enhanced as compared to the Schiff bases.

### 2.7. DNA Binding Studies

**Figure 6:**
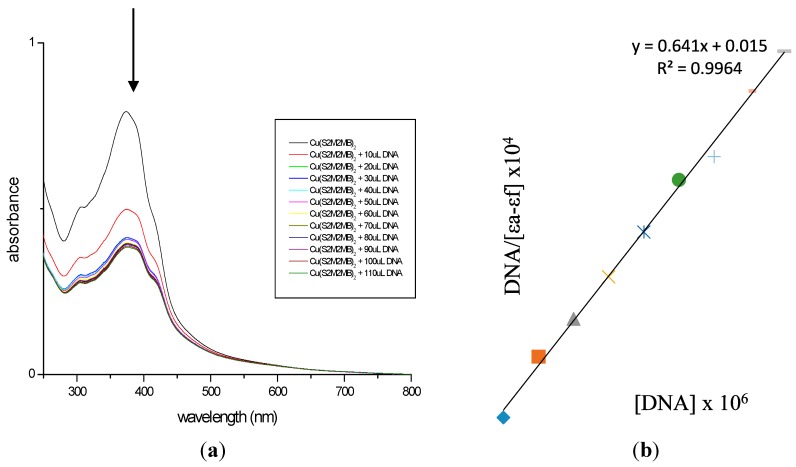
(**a**) Electronic absorption spectrum of **3**; (**b**) Plot of [DNA]/ε_a_−ε_f_ *vs.* [DNA] for absorption titration of DNA with **3**. (The arrow indicates the change in absorbance in tandem with increasing DNA concentration.).

**Figure 7:**
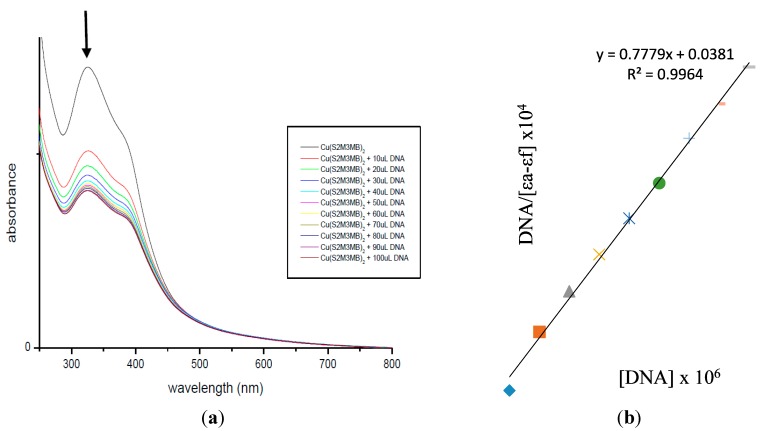
(**a**) Electronic absorption spectrum of **6**; (**b**) Plot of [DNA]/ε_a_−ε_f_ *vs.* [DNA] for absorption titration of DNA with **6**. (The arrow indicates the change in absorbance in tandem with increasing DNA concentration.).

The interaction between Cu(II) complexes (**3** and **6**) and calf thymus DNA (CT-DNA) was studied using electronic absorption titration [36]. Hypochromism and red-shifts indicate strong stacking interaction between the DNA double helix strand and an aromatic chromophore [36,37,38]. The electronic absorption spectra for the Cu(II) complexes (**3** and **6**) recorded in the absence and presence of CT-DNA are shown in Figure 6 and Figure 7.

Each displayed two intense absorption bands attributed to the LMCT and intra-ligand (π → π*) transitions of an aromatic chromophore in the regions 322–392 and 263–282 nm, respectively. The decreasing energies of π → π* indicated that the π* orbital of the ligands on the complexes were coupled with a π orbital of the DNA base pairs. Therefore, this interaction was manifested by hypochromism and red-shifts [39]. In order to compare the binding strengths of the complexes to CT-DNA, the binding constants were determined quantitatively. The K_b_ obtained for complexes 3 and 6 were 4.11 × 10^5^ and 2.04 × 10^5^ M^−1^, respectively, suggesting both the complexes have good binding affinities due to π → π* stacking interactions between the respective complex and the base pairs of DNA [38,40,41]. Complex 3 has the higher binding constant which correlates with it having the lowest IC_50_ value among the compounds assayed. The greater potency of 3 against cancer cells arises from its ability to block the enzyme that bind to the nitrogenous bases of DNA or RNA [42].

## 3. Experimental Section

### 3.1. Chemicals

All chemicals and solvents were of analytical grade and were used as received without further purification. IR spectra were recorded in KBr pellets on a Perkin-Elmer1750X FTIR spectrophotometer (Waltham, MA, USA) in the range of 4000–280 cm^−1^. C, H, N and S elemental analyses were carried out using a LECO CHNS-932 instrument (St. Joseph, MI, USA). Metal determinations were carried out using a Perkin-Elmer Plasma 1000 Emission Spectrometer (Waltham, MA, USA). Molar conductivities of 10^−3^ M solutions of the metal complexes in DMSO were measured at 27 °C using a Jenway 4310 conductivity meter (Dunmow, Essex, UK) and a dip-type cell with a platinized electrode. Electronic spectra were recorded on a Shimadzu UV-2501 PC recording spectrophotometer (1000–200 nm) (Tokyo, Japan). Magnetic susceptibilities were measured with a Sherwood Scientific MSB-AUTO magnetic susceptibility balances at 298 K (Sherwood Scientific, Cambridge, UK). Melting points were determined using an Electrothermal digital melting point apparatus (Electrothermal Engineering Ltd., Essex, UK).

### 3.2. Syntheses

#### 3.2.1. *S*-2-Methybenzyldithiocarbazate (S2MBDTC)

Following a procedure adapted from Ravoof *et al.* [43], potassium hydroxide (11.4 g, 0.2 mol) was dissolved in ethanol (70 mL, 90%). Then, the hydrazine hydrate (10 g, 0.2 mol) was added and the mixture was maintained at 0 °C in an ice-salt bath. Carbon disulfide (15.2 g, 0.2 mol) was added drop-wise with vigorous stirring (750 rpm) over a period of 1 h. The two layers that formed were separated using a separating funnel. The light-brown lower layer was dissolved in 40% ethanol (60 mL) below 5 °C. The mixture was kept in an ice-bath and 4-methylbenzyl chloride (26.5 mL, 0.2 mol) was added drop-wise with vigorous stirring. The sticky white product, which formed S4MBDTC, was filtered and left to dry overnight in a desiccator over anhydrous silica gel. Yield: 68%. Mp 170–171 °C (lit. 171.2 °C).

#### 3.2.2. *S*-2-Methylbenzyl-β-*N*-(2-benzyl-2-methoxymethylene)dithiocarbazate (NS^1^) (**1**)

S2MBDTC (2.12 g, 0.01 mol) was dissolved in hot acetonitrile (100 mL). An equimolar amount of 2-methoxybenzaldehyde (1.36 g, 0.01 mol) was added to the S2MBDTC solution and the mixture was stirred and heated to reduce the original volume of the mixture. The mixture was then stirred under room temperature until a precipitate formed, which was washed with ice-cold ethanol. Compounds were recrystallized from ethanol and dried over silica gel.

#### 3.2.3. *S*-2-Methylbenzyl-β-*N*-(2-benzyl-3-methoxymethylene)dithiocarbazate (NS^2^) (**2**)

S2MBDTC (2.12 g, 0.01 mol) was dissolved in hot acetonitrile (100 mL). An equimolar amount of 3-methoxybenzaldehyde (1.36 g, 0.01 mol) was added to the S2MBDTC solution and the mixture was stirred and heated to reduce the original volume of the mixture. The mixture was then stirred under room temperature until a precipitate formed, which was washed with ice-cold ethanol. Compounds were recrystallized from ethanol and dried over silica gel.

#### 3.2.4. Metal Complexes Derived from Ni(II) Acetate, Cu(II) Acetate, and Zn(II) Acetate

Schiff base (0.001 mol) in hot acetonitrile (50 mL) was added to the metal salt (0.0005 mol) in ethanolic solution (30 mL). The mixture was heated and stirred to reduce the volume of the solution. Precipitation occurred once the mixture cooled to the room temperature. The precipitate then was filtered and dried over silica gel. The complexes were recrystallized using methanol to purify the complexes. The metal salts used were: nickel (II) acetate tetrahydrate, zinc(II) acetate dihydrate, and Cu(II) acetate monohydrate.

### 3.3. X-ray Crystallography

The X-ray diffraction measurements for yellow crystals of **1** (0.24 × 0.29 × 0.37 mm) and **2** (0.20 × 0.31 × 0.37 mm) were performed at 100 K on an Oxford Diffraction Gemini CCD diffractometer [44] using CuKα radiation (λ = 1.5418 Å) and ω scans so that θ_max_ was 71.4°. The structures were solved by direct methods and refined on *F*^2^ (anisotropic displacement parameters, C-bound H atoms in the riding model approximation and a weighting scheme of the form *w* = 1/[σ^2^(*F*_o_^2^) + *aP*^2^ + *bP*] where *P* = (*F*_o_^2^ + 2*F*_c_^2^)/3) using the SHELXL2014 package of programs [45] through the WinGX Interface [x3]. The N-bound hydrogen atoms were refined with the N–H distance constraint 0.88 ± 0.01 Å. Crystal data and refinement details are collated in Table 6. The molecular structures shown in Figure 1 and Figure 2 were drawn with 70% displacement ellipsoids [46]. The overlay diagram, Figure 3, was drawn with QMol [47] and the crystal packing diagrams with DIAMOND [48]. Crystallographic data for **1** and **2** have been deposited with the Cambridge Crystallographic Data Centre as supplementary publication numbers CCDC 1035013 and 1035014.

### 3.4. Cytotoxic Assay

The MCF-7 (estrogen receptor-positive human breast cancer) and MDA-MB-231 (estrogen receptor-negative human breast cancer) cell lines were used in this study (ATCC, Manassas, VA, USA). The cells were cultured in RPMI-1640 (High glucose) (Sigma, St. Louis, MO, USA) medium supplemented with 10% fetal calf serum. The cytotoxic activity was determined using the microtitration of 3-(4,5-dimethylthiazol-2-yl)-2,5-diphenyltetrazolium bromide (MTT) assay (Sigma) as previously reported [49]. Untreated cells were included for each assay as the negative control. The standard breast cancer chemotherapeutic, Tamoxifen, was used as the standard positive control. Cytotoxicity levels were expressed as IC_50_ values, *i.e.*, the concentration of compound that results in 50% cell death as compared to the negative controls *in vitro*.

**Table 6:** Crystallographic data and refinement details for **1** and **2**.

Compound	1	2
Formula	C_17_H_18_N_2_OS_2_	C_17_H_18_N_2_OS_2_
Formula weight	330.45	330.45
Crystal system	monoclinic	monoclinic
Space group	*P*2_1_/*n*	*P*2_1_/*c*
*a*/Å	14.4313(2)	17.6572(6)
*b*/Å	6.0887(1)	5.2747(2)
*c*/Å	18.4462(3)	19.3205(7)
β/°	97.074(2)	114.736(4)
*V*/Å^3^	1608.49(4)	1634.33(11)
*Z*	4	4
*D*_c_/g·cm^−3^	1.365	1.343
*F*(000)	696	696
μ/mm^−1^	3.017	2.969
Measured data	19,873	10,986
θ range/°	3.7–71.4	4.6–71.4
Unique data	3128	3151
Observed data (*I* ≥ 2.0σ(*I*))	3058	2965
*R*, obs. data; all data	0.033; 0.034	0.033; 0.035
*a*, *b* in weighting scheme	0.057, 0.763	0.052, 0.787
*R*_w_, obs. data; all data	0.089; 0.089	0.086; 0.088
Residual electron density peaks/e Å^3^	0.31, −0.42	0.43, −0.30

### 3.5. DNA Binding Studies

The DNA binding experiments were carried out at 25 °C. DNA concentration per nucleotide was determined using the molar absorption coefficient (6600 M^−1^·cm^−1^) at 260 nm [50]. Absorption titration experiments were performed maintaining the concentration of the metal complex solution at 50 µM and gradually increasing the concentration of CT-DNA. The compounds were dissolved in DMSO and Tris-HCI buffer (50:50) containing (5 mM Tris, pH 7.1, 25 mM NaCl) [39] at room temperature (25 °C). The solutions were scanned over the 250–800 nm range. Absorbance values were recorded 10 min after the addition of DNA solution. The binding constant, K_b_ was determined, using the Equation:

(1) $$\left[DNA]/(\varepsilon_{a}\;-\;\varepsilon_{f}\right)\;=\;\left[DNA]/(\varepsilon_{b}\;-\;\varepsilon_{f}\right)\;+\;1/K_{b}(\varepsilon_{a}\;-\;\varepsilon_{f})$$

where [DNA] is the concentration of DNA in base pairs, εa corresponds to the apparent molar extinction coefficient Aabs/[M], ε_f_ corresponds to the extinction coefficient for the free metal [M] complex and ε_b_ corresponds to the extinction coefficient for the fully bound metal complex [39,50,51,52].

## 4. Conclusions

Schiff bases **1** and **2** act as bidentate ligands coordinating through the sulfur and nitrogen atom forming metal complexes with Cu(II), Ni(II) and Zn(II). The coordination of these structurally similar Schiff bases to different metal ions was confirmed by physicochemical techniques and various spectral studies. X-ray crystallography showed isomeric **1** and **2** to have similar structures, each with a conformation akin to the letter L. The cytotoxic studies of all the compounds were carried out against MCF-7 (estrogen receptor-positive human breast cancer) and MDA-MB-231 (estrogen receptor-negative human breast cancer) cell lines and their IC_50_ values were determined. The Cu(II) complexes **3** and **6** were found to be moderately active against both cancer cell lines but the other complexes were inactive. In agreement with cytotoxic assay data, the DNA binding studies indicated that complexes **3** and **6** bound to CT-DNA with a good binding affinity, K_b_ = 10^5^ M^−1^.
